# Multimodal fluorescence molecular imaging for *in vivo* characterization of skin cancer using endogenous and exogenous fluorophores

**DOI:** 10.1117/1.JBO.22.6.066007

**Published:** 2017-06-14

**Authors:** Jessica P. Miller, LeMoyne Habimana-Griffin, Tracy S. Edwards, Samuel Achilefu

**Affiliations:** a Washington University School of Medicine, Mallinckrodt Institute of Radiology, Optical Radiology Laboratory, St. Louis, Missouri, United States; b Washington University in St. Louis, Biomedical Engineering, St. Louis, Missouri, United States

**Keywords:** squamous cell carcinoma, nonmelanoma skin cancer, tumor, near-infrared, autofluorescence, fluorescence molecular tomography, depth, imaging, fluorescence lifetime imaging

## Abstract

Similarity of skin cancer with many benign skin pathologies requires reliable methods to detect and differentiate the different types of these lesions. Previous studies have explored the use of disparate optical techniques to identify and estimate the invasive nature of melanoma and basal cell carcinoma with varying outcomes. Here, we used a concerted approach that provides complementary information for rapid screening and characterization of tumors, focusing on squamous cell carcinoma (SCC) of the skin. Assessment of *in vivo* autofluorescence lifetime (FLT) imaging of endogenous fluorophores that are excitable at longer wavelengths (480 nm) than conventional NADH and FAD revealed a decrease in the short FLT component for SCC compared to normal skin, with mean values of 0.57±0.026  ns and 0.61±0.021  ns, respectively (p=0.004). Subsequent systemic administration of a near-infrared fluorescent molecular probe in SCC bearing mice, followed by the implementation of image processing methods on data acquired from two-dimensional and three-dimensional fluorescence molecular imaging, allowed us to estimate the tumor volume and depth, as well as quantify the fluorescent probe in the tumor. The result suggests the involvement of lipofuscin-like lipopigments and riboflavin in SCC metabolism and serves as a model for staging SCC.

## 1 Introduction

Skin cancer is the most common cancer worldwide with the World Health Organization estimating 2 to 3 million cases each year. The disease comprises many types, including basal cell and squamous cell carcinoma (SCC), which are forms of nonmelanoma skin cancer (NMSC). The incidence of NMSC is increasing, underscoring the importance of early detection and diagnosis.^1^^–^^3^ Skin cancer is often detected visually, followed by the use of biopsy for histopathological validation. Visible signs of skin cancer can include changes in skin color, roughness, a raised area, or a wound that does not heal. However, the disease can be more subtle, in some cases presenting as an area that is pruritic with altered sensation.^4^ In a subset of cases, there is no obvious border to a skin cancer tumor which can lead to the incomplete resection that occurs in as high as 15% of cases.^5^ Because of the diverse appearance of skin cancers and the sometimes obscure tumor border, a reliable detection method would be beneficial. The use of optical imaging methods to increase the accuracy of identifying tumors and their boundaries is attractive because of the low cost, portable instrumentation, and the ability to provide real-time feedback.

Because SCC is on the surface of the body, imaging and analyzing a region of skin could prove effective in reliably identifying the disease. Malignant tumors are known to induce significant changes in the size of cellular organelles, pigmentation, and the concentrations or ratios of endogenous fluorophores compared to normal skin. As a result, light scattering and fluorescence intensity measurements have been used to characterize healthy and pathologic skin conditions in small animals and humans.^6^^–^^11^ Autofluorescence imaging is widely used to characterize skin tissue due to the presence of a heterogeneous mixture of endogenous fluorophores, including NADH, elastin, collagen, and flavins.^12^ To minimize imaging artifacts, improve the reproducibility and reliability of diagnostic information, and reduce errors caused by the contribution of a myriad of endogenous fluorophores to the intensity measurements, autofluorescence lifetime (FLT) focusing on NADH and FAD has been explored^13^^,^^14^ for melanoma^15^ and basal cell carcinoma.^16^ Previous studies have demonstrated that fluorescence lifetime imaging microscopy can be used to differentiate NMSC from uninvolved tissue.^16^^,^^17^ However, SCC exhibits a diverse array of gross pathological features, necessitating the survey of a larger tissue area than what is typically accessible by microscopic methods. In addition, recent studies have shown that some endogenous fluorophores, such as lipofuscins, lipofuscin-like lipopigments, and riboflavin, enhance the intracellular fluorescence of malignant tumors upon excitation at 450 to 500 nm.^18^^–^^20^

For staging, knowing the extent of the penetration of the disease is important. Although the FLT can identify skin lesions, visible light can only penetrate a few millimeters into the tissue, limiting tissue analysis to superficial regions. Conversely, near-infrared (NIR) light is less absorbed by the tissue and can penetrate to greater depths, allowing the use of NIR fluorescent molecular probes to differentiate tumors from healthy tissue when they accumulate preferentially in the cancer.^21^^,^^22^ A variety of methods are available for delivering the molecular probes to tumors, including reliance on the enhanced permeation and retention effect, conjugation to an antibody or a peptide that targets cancer cells, and constructing nanoparticles containing tumor-homing moieties.^23^

In this study, we explored the use of *in vivo* FLT imaging of biomarkers that are excitable at longer wavelengths than conventional NADH and FAD for distinguishing NMSC from surrounding uninvolved skin. Image analysis of fluorescence intensity data from planar imaging and NIR fluorescence molecular tomography (FMT) allowed us to determine the tumor extent and quantify the molecular probe concentration in SCC.

## 2 Methods

### 2.1 Animal Model Development

All studies were in compliance with the Washington University Animal Welfare Committee’s requirements for the care and use of laboratory animals in research. We used an orthotopic model of SCC-12 (human cutaneous SCC) to develop methods for skin cancer identification and characterization.^24^ SCC-12 cells were cultured using Dulbecco's modified Eagle's medium supplemented with 10% fetal bovine serum and 1% penicillin streptomycin in a humidified incubator at 37°C. We injected $2.5\times 10^{6}\text{ cells}$ into the intradermal compartment of 6-week old female athymic nude mice (n=5) in the bilateral shoulder and flank regions by placing the needle just below the surface of the skin. Tumors were allowed to grow for 3 weeks before imaging. In some cases, the tumors did not graft, and these regions were not included in the study.

### 2.2 Autofluorescence Imaging

FLT imaging was conducted using the Optix MX3 system (ART Advanced Research Technologies, Montreal, California) with excitation and emission wavelengths at 480 and 535 nm, respectively. Full quantitative analysis was performed to obtain the FLT for each pixel.

### 2.3 Near-Infrared Fluorescence In Vivo Imaging

Our lab has previously reported on an NIR fluorescent probe that selectively accumulates in tumors *in vivo*.^25^ This probe, cypate-peptide derivative (cypate-GRD), has demonstrated efficacy in a number of different tumor types, but it has not yet been explored in skin cancer. The compound was injected via the tail vein, at a dose of 0.40  mg/kg, suspended in 100  μL of phosphate-buffered saline. The injected amount was selected by identifying a dose that would allow for adequate visualization of the tumors without increasing the background signal. Animals (n=4) were imaged at 24-h postinjection using the Pearl Small Animal NIR fluorescence imaging system (LICOR Biosciences, Nebraska), λex/em 785/820  nm. Fluorescence and reflectance images were obtained using the FMT 4000 system (PerkinElmer, Inc., Massachusetts) λex/em 790/>805  nm.

### 2.4 Near-Infrared Fluorescence Ex Vivo Imaging

Excised tumor tissues were flash-frozen in optimum cutting temperature compound (Tissue Tek, California) and stored at −20°C. The tumors were sliced at a thickness of 10  μm (Cryocut 1800, Illinois). H&E staining of excised tumor and surrounding tissues was used for histologic validation of tissue types. Microscopy was performed with an Olympus BX51 upright microscope (Olympus America, Pennsylvania). An NIR filter (U-N41130 Chroma Technology Corp, Vermont) was used for excitation at 775±25  nm and emission at 845±27  nm.

### 2.5 Near-Infrared Tumor Characterization

Tumor volume was estimated using a gradient-based tumor volume algorithm.^26^ For depth estimation, we utilized the fluorescence and reflectance images obtained using the FMT system.^27^ We incorporated our fluorescence gradient-based approach to the FMT method to define the tumor regions of interest (ROIs) for analysis. The fluorescence wavelength was 810 nm ($\lambda_{2}$), and the reflectance wavelength was the excitation light wavelength of 790 nm ($\lambda_{1}$). The following equations were used to calculate the depth as a function of the natural log of the ratio of the intensity at the two wavelengths:

$$\ln(\Gamma )=(\frac{1}{\delta^{\lambda 2}}-\frac{1}{\delta^{\lambda 1}})\times d+\ln(\frac{D^{\lambda 2}}{D^{\lambda 1}}),$$ (1)

$$D=\frac{1}{3(\mu_{a}+\mu_{s}^{\prime })},$$ (2)

$$\delta =\sqrt{\frac{D}{\mu_{a}}},$$ (3)

ln(Γ)=m×d+b, (4)

$$d=\frac{\ln(\Gamma )-b}{m},$$ (5)

where Γ is the ratio of the fluorescence signal intensity to the reflectance signal intensity, δ is the penetration depth, D is the diffusivity based on the optical properties at each wavelength ($\lambda_{1}$ and $\lambda_{2}$), and d is the depth. The general form of Eq. (4) can be rearranged and solved for depth using Eq. (5). The slope and y-intercept were calculated as m and b, respectively.

The input tissue optical parameters for Eqs. (2) and (3), $\mu_{\alpha }$ and $\mu_{S}^{\prime }$, were estimated at our wavelengths of interest (790 and 810 nm) from a model developed by Jacques.^28^ Using this model, we assumed the fraction of melanin as 3.8% for mice (average for a light-skinned adult human) and the model predicted $\mu_{\alpha 790}=5.867$, $\mu_{\alpha 810}=5.208$, $\mu_{S790}^{\prime }=14.142$, and $\mu_{S810}^{\prime }=12.941\;{\mathrm{cm}}^{-1}$. When these values were used in Eq. (1) through Eq. (4), m=−1.927 and b=0.098. We used these parameters to solve for depth pixel-by-pixel using Eq. (5). Because this method was susceptible to variability in the y-intercept value (b), the maximum spread of the depths within the tumor region was used to represent the depth as opposed to the absolute values obtained.

Three-dimensional (3-D) tumor images were obtained using the FMT system with 2-mm source density. Reconstruction and image analyses were performed using TrueQuant™ software (PerkinElmer, Inc., Massachusetts). Fluorescence quantification of our NIR fluorophore was based on a concentration calibration using the calibration phantom provided with the system. Rectangular prism ROIs were drawn around tumors of 3-D reconstructed images, using the planar fluorescence images for guidance.

## 3 Results and Discussion

**Fig. 1:**
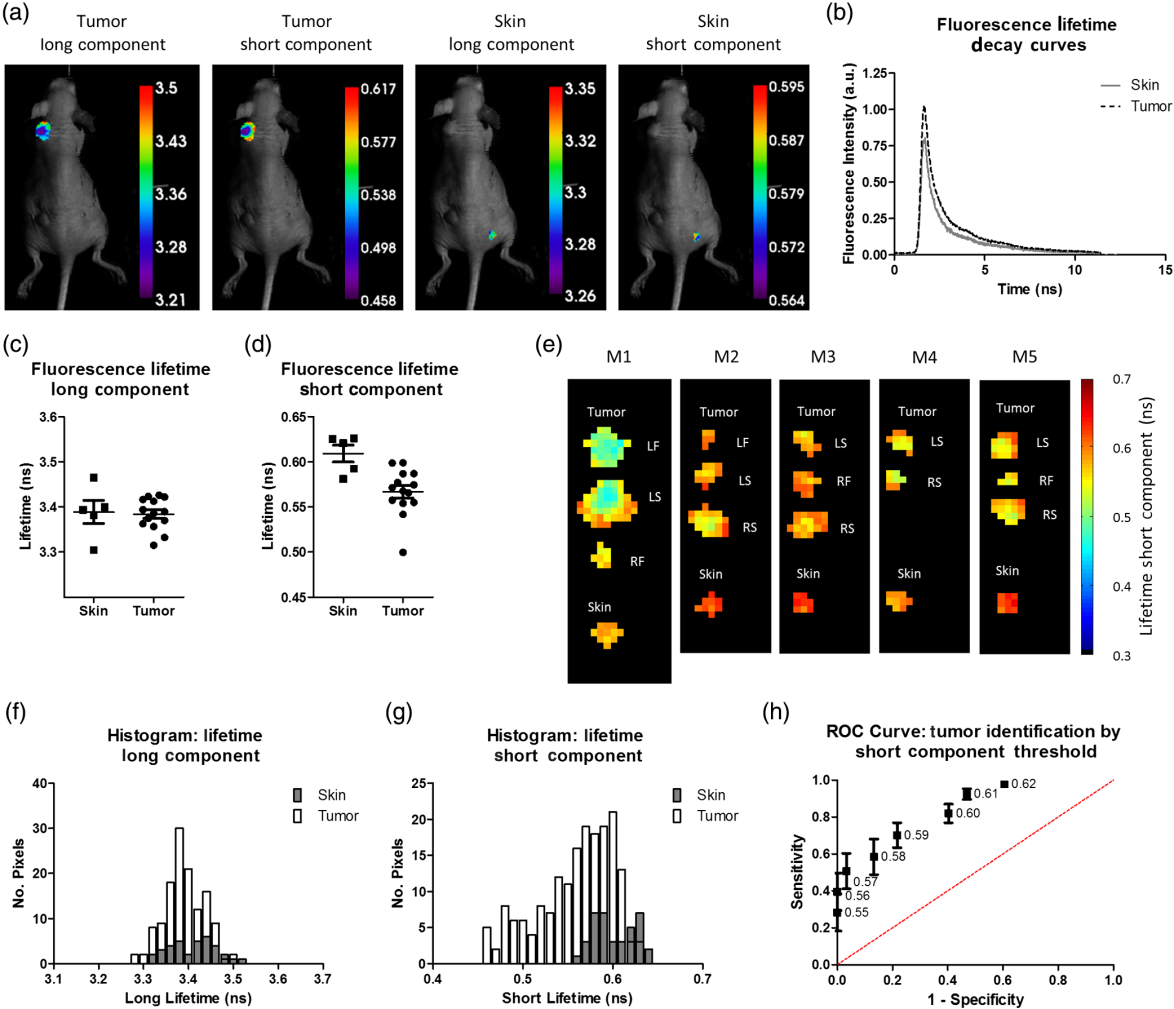
(a) Representative mouse images showing the lifetime of a tumor region and an uninvolved skin region. A total of 5 mice with 14 tumors were analyzed. (b) Representative FLT decay showing a steep initial decay then a slower decay at longer times. (c) Average FLT long component for skin and tumor showing no difference. (d) Average FLT short component for skin and tumor showing a lower lifetime for the tumor, p=0.004. (e) Lifetime maps for each pixel for tumors and skin. Tumors exhibited shorter lifetimes with the more central region containing the shortest lifetimes. Tumor locations are indicated as left flank (LF), left shoulder (LS), right flank (RF), and right shoulder (RS). (f) Histogram of long lifetime component for each pixel showing no difference. (g) Histogram for short lifetime component showing that shorter values are unique to tumors. (h) ROC curve showing the sensitivity and specificity for tumor identification using different short lifetime thresholds (the data point labels).

We chose 480-nm excitation to image due to the abundance of endogenous fluorophores, such as flavins, lipofuscins, and lipofuscin-like, in this spectral range.^19^ FLT images of the tumor regions and a skin region on the mouse flank were determined [Fig. 1(a)]. Analysis of the fluorescence intensity decays for each region indicated the presence of two different time constants in the overall decay [Fig. 1(b)]. We found that a single-component fit did not fit the shape of the decay. To most accurately represent the decay, a fit that represented each of the fluorescence molecules present would be ideal, however, less practical. By using a two-component fit, we were able to adequately fit the FLT decay; however, some variation between samples remained due to the heterogeneous composition of the tissue itself.

Because we were interested in understanding if FLT could be applied to a macroscopic image of the tumor, we compared the average values of our ROIs. Our assessment of the average FLT of the long component for each ROI did not reveal any difference between the tumors and skin regions [Fig. 1(c)]. In contrast, the analysis of the average lifetime of the short component revealed a difference between regions containing tumors and skin, with a mean lifetime of 0.57±0.026  ns and 0.61±0.021  ns, respectively [Fig. 1(d)]. This difference was significant at p=0.004. One of the short component FLT values was lower than the rest in the tumor group. To ensure that the significant difference between the skin and tumor was not due to this point, we recalculated the data for statistical comparison. The p-value with this point removed was also significant at p=0.002. We attributed this FLT value to the often-observed heterogeneity in biological assays or the redox state of the cancer at the time of measurement.

We created lifetime pixel maps using the short lifetime, and we observed that the shorter lifetimes were associated with the more central regions of the tumors [Fig. 1(e)]. Short lifetime values within the tumors that were closer to the skin values were located around the edges, which implies that these areas were more similar to the surrounding skin tissue. Using each of the pixel values to create histograms for the long and short components of the lifetime, we confirmed that the long lifetimes had the same distribution for tumors and skin, while the short lifetimes exhibited values that were unique for the tumors [Figs. 1(f) and 1(g)]. These data demonstrate a spatial distribution of the short lifetime values within the ROIs.

We investigated the sensitivity and specificity of using the short lifetime component to delineate tumors from skin by setting a threshold [Fig. 1(h)]. The mean short lifetime for each ROI was more sensitive than specific at all thresholds because the receiver operating characteristic (ROC) curve consistently favored higher sensitivity values than the corresponding specificity value for each point. The spatial information appears to have an impact on the short lifetime, with shorter lifetimes more centrally located within the tumor. Hence, combining the short lifetime data with spatial information could enhance both sensitivity and specificity of the method. Literature reports have identified lipofuscins, lipofuscin-like lipopigments, and riboflavins as the major fluorescent species at the wavelengths we have explored.^18^^–^^20^ These biomolecules are actively involved in cellular metabolism. Miranda-Lorenzo et al.^20^ identified riboflavin as the primary autofluorescent moiety in the 480-/535-nm excitation/emission region. The authors attributed the increase in riboflavin autofluorescence to cells with high proliferation potential. However, we do not know at his time how the metabolic processes in the tumor affected the short FLTs in cancer and healthy skin. Probably, response of the endogenous fluorophores to pH, oxygenation status, and redox state of cancer differs significantly from those of healthy tissue.

**Fig. 2:**
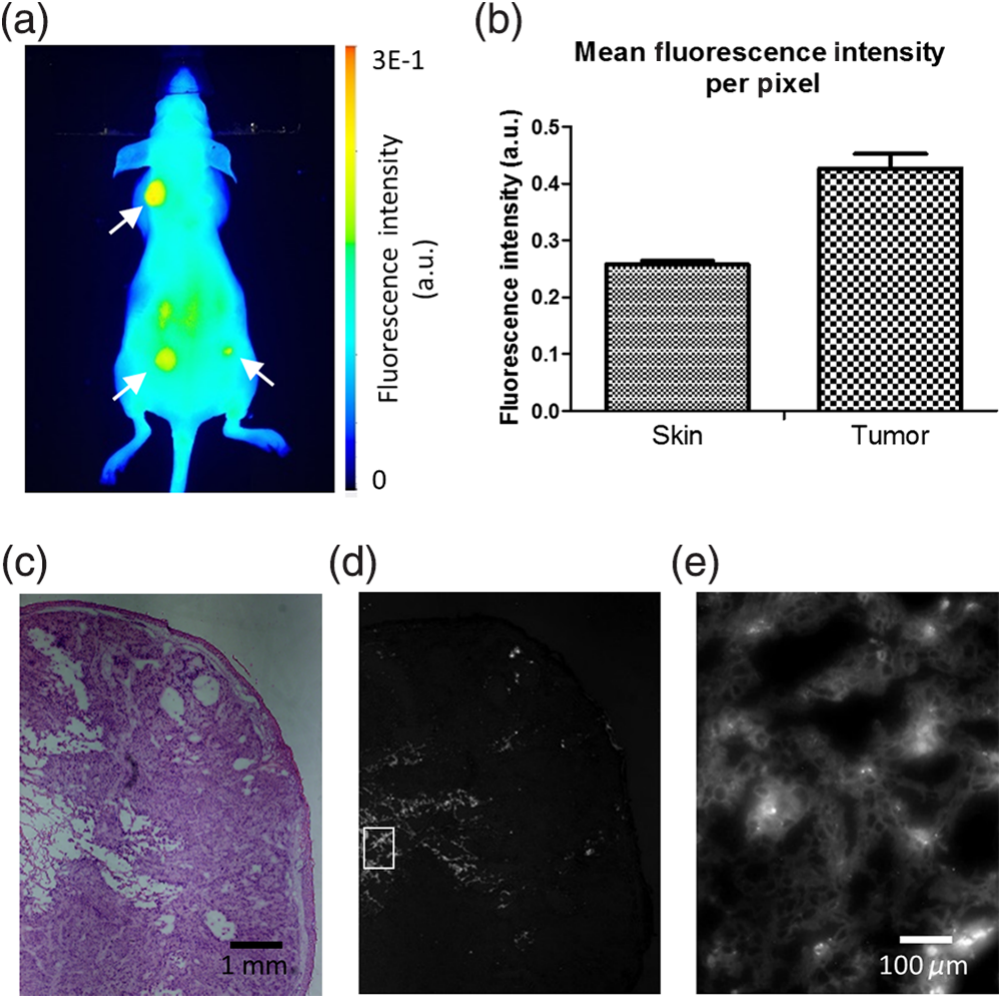
(a) Representative image of NIR fluorescent probe *in vivo* showing tumor contrast (white arrows). (b) Quantification of skin regions versus tumor regions with tumors exhibiting higher fluorescence. A total of three tumor regions and three skin regions were analyzed. (c) H&E staining of an SCC tumor was analyzed at 4× magnification. (d) Fluorescence distribution of the fluorescent probe within the tumor. (e) 40× magnified view of fluorescent probe with cells within the tumor.

The autofluorescence method enabled us to image the superficial aspects of the tumors. However, the shallow depth penetration of visible light in tissue limited the information that we could obtain. To gain insight into deeper tumor characteristics, we injected an NIR fluorophore and allowed it to accumulate in the tumors [Fig. 2(a)]. Quantification of the *in vivo* fluorescence intensity showed that the tumors exhibited a higher signal (0.42±0.044) than the skin regions (0.26±0.012), p=0.003. [Fig. 2(b)] After concluding the *in vivo* study, we euthanized a mouse and confirmed the presence of our fluorophore in the tumor tissue. Figures 2(c) and 2(d) show the tumor histology and fluorophore distribution within the tumor, respectively. A section of the tumor was magnified to visualize the fluorophore along with cells within the tumor [Fig. 2(e)].

**Fig. 3:**
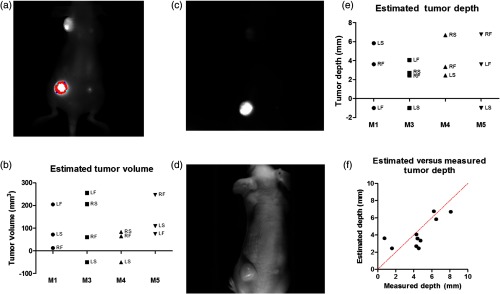
(a) Representative gradient-based tumor volume algorithm NIR fluorescence images showing the tumor outline used to estimate the tumor volume. (b) Estimated tumor volume by mouse and tumor (M2 was not injected with the fluorescent probe). A total of 4 mice and 13 tumors were analyzed, 11 tumors returned results. Tumor locations are indicated by LF, LS, RF, and RS. (c) Fluorescence image used for the fluorescence–reflectance depth imaging analysis. (d) Reflectance image used for the fluorescence–reflectance depth imaging analysis. (e) Depth estimates using the fluorescence–reflectance method by mouse and by tumor. A total of 4 mice and 13 tumors were analyzed, 10 tumors returned results. (f) Estimated versus measured depth for each of the tumors where a depth estimate was successfully obtained.

We explored the tumor extent using the NIR signal as a guide. Figure 3(a) shows a representative NIR fluorescence image of a tumor-bearing mouse captured using a planar imaging system. Images were analyzed for tumor volume using a previously described gradient-based approach^26^ and are shown for each tumor [Fig. 3(b)]. Tumors that had inadequate contrast for the algorithm to calculate a volume were represented with a negative value in the plot. The tumor volume estimation method used the equation $V=0.5\times L\times W^{2}$, where V is the tumor volume, L is the tumor length, and W is the tumor width. This equation used the tumor width as a surrogate for understanding the tumor depth. Because of this assumption, the translation of this approach to broader applications outside of small animal imaging is limited. To understand the extent of the tumor more directly, the depth dimension would need to be calculated rather than using the width as a surrogate for depth.

To accomplish depth estimation, we used a fluorescence-to-reflectance ratio method to determine the extent of tumor penetration. Swartling et al.^29^ used this approach to calculate the depth for a point-like fluorescence source, and Kolste et al.^27^ expanded this method to depth estimation using planar imaging. Using the FMT imaging system, we obtained fluorescence [Fig. 3(c)] and reflectance [Fig. 3(d)] images of the mice. We defined tumor versus nontumor ROIs using the same gradient-based approach for tumor isolation that we used to obtain the tumor volume. For pixels defined as tumor, we divided the fluorescence signal by the reflectance signal and then utilized the natural log of the ratio for the depth estimation. This method required knowledge of the tissue optical parameters at the wavelengths of interest. We estimated these values using a previously developed model for tissue property estimation as a function of wavelength.^28^ We found that the depth estimation method was very sensitive to the parameters that dictated the y-intercept value of the curve fit, which were in turn impacted by the optical parameters selected. To stabilize the depth estimation output, we calculated the difference in depth between the maximum and minimum depth values within the tumor ROI and recorded this value as the estimated depth [Fig. 3(e)]. Saturated pixels were excluded from the analysis, and tumors that returned no depth values were represented as negative values in the plot. The method did not return values in two tumors [M1 left flank (LF) and M5 left shoulder (LS)] due to image saturation and one tumor (M3 LS) due to inadequate fluorescence signal. The estimated tumor depths were compared to the caliper measured tumor depths [Fig. 3(f)]. The red line shows the ideal case of correlation between the method estimates and the measured depths. While there was some difference between the estimated result and the idealized values, the average deviation from the caliper measurement was ±1.244  mm down to an imaging depth of 8 mm.

**Fig. 4:**
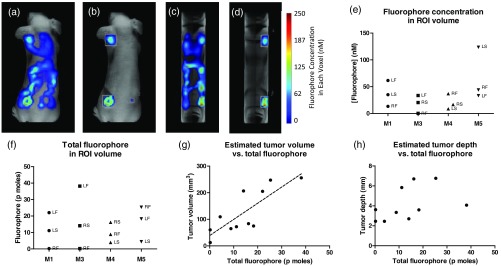
(a) 3-D FMT image showing whole body distribution of the NIR fluorescence signal. Tumors exhibited a higher uptake than the surrounding tissue. The mouse digestive tract was visible due to the fluorescence from the chow. (b) Tumors enclosed in ROIs to remove fluorescence from the digestive tract. The concentration of fluorophore shown in 3-D within the tumor ROIs. (c) Sagittal view of NIR fluorescence signal. (d) Sagittal view of tumors in ROIs. (e) Quantification of fluorophore amount per voxel in each tumor ROI. A total of 4 mice and 12 tumors were analyzed. Tumor locations are indicated by LF, LS, RF, and RS. (f) Quantification of total fluorophore amount in each ROI. (g) Estimated tumor volume versus fluorophore amount showing a correlation, p=0.003. A total of 11 tumors had values for both the fluorophore amount and estimated tumor volume. (h) Estimated tumor depth versus fluorophore amount not correlated. A total of 10 tumors had values for both the fluorophore amount and estimated tumor depth.

We then employed 3-D NIR tomography to understand the extent of tumors. Figures 4(a) and 4(c) show a mouse with the 3-D distribution of the NIR fluorescent probe in both the coronal and sagittal views. Although the tumors were visible, there was also high signal from the intestinal tract as mouse chow has NIR fluorescence. To remove background fluorescence, ROIs were drawn around each tumor [Figs. 4(b) and 4(d)]. Figure 4(e) shows the fluorophore concentration in each tumor based on the ROI volume, and Fig. 4(f) shows the total amount of the fluorophore within the ROI. We compared the estimated tumor volume to the total fluorophore amount to determine if the probe accumulation was related to tumor volume [Fig. 4(g)]. We found that they were correlated with a Spearman’s correlation coefficient of 0.82, p=0.003. However, the probe accumulation was not correlated with the estimated tumor depth [Fig. 4(h)].

## 4 Conclusions

Noninvasive imaging has an advantage over direct tumor measurement and evaluation in that it allows for understanding the functional and structural behavior of a tumor. Imaging also allows for the retrospective analysis of images to extract additional information and track therapeutic efficacy. We evaluated SCC using a number of imaging modalities to both identify and characterize the disease *in vivo*. The FLT imaging results were consistent with previous work which found that basal cell carcinoma, another form of NMSC, had shorter FLTs as compared to uninvolved tissue using NADH and FAD as biomarkers.^16^ Although the differences in the short FLT between cancer and healthy skin were statistically significant and sufficient to distinguish tumor from surrounding tissue, practical application of the technique would require the development of optical systems with high temporal and spatial resolution to improve the detection accuracy. A key component to the successful implementation of this approach is to establish a reliable FLT threshold for specific tumor types. With recent advances in the development of devices that allow for *in vivo* assessment of FLT,^30^ we expect that our findings can be used to identify tumor versus uninvolved tissue *in vivo*.

We used an NIR fluorescent probe to capture the extent of the tumor by estimating the tumor volume, which can be used to track a therapeutic response. Further, we demonstrated that NIR fluorescence planar imaging could be used to estimate tumor depth with relative agreement to the measured values. The relative ease of our method could overcome the challenges of assessing tumor depth noninvasively. Finally, we demonstrated the ability of FMT to capture the amount of a fluorescent probe in the tissue. By incorporating fluorescent probes into drugs, or the use of drugs that emit light, FMT could be used to track the amount of a drug that reaches a target, particularly for therapies such as photodynamic therapy, where the amount of drug present can impact the desired light dose within a region.

The summation of this work provides a framework for the optical interrogation of skin cancer. Each of our methods was conducted *in vivo* in a living mouse, which makes it ideal for longitudinal studies tracking therapeutic response. Use of FLT allowed us to identify SCC tumors without the need for an exogenous imaging agent. Because light-based methods are cost effective and can be implemented in real time, the optical imaging techniques described in this paper can be used to screen and stage NMSC lesions in human subjects. Our results suggest that the integration of different optical methods could improve the accuracy of identifying, tracking, and phenotyping tumors, in hopes of improving existing diagnoses and therapies. This study provides a model for screening SCC with endogenous fluorophores, staging the disease with exogenous fluorescent molecular probes, and quantifying the amount of a drug-probe combination for future monitoring of therapeutic drug dosing.
